# Island survivors: population genetic structure and demography of the critically endangered giant lizard of La Gomera, *Gallotia bravoana*

**DOI:** 10.1186/s12863-014-0121-8

**Published:** 2014-11-25

**Authors:** Elena G Gonzalez, Ivania Cerón-Souza, José A Mateo, Rafael Zardoya

**Affiliations:** New address: Departamento de Biodiversidad y Biología Evolutiva, Museo Nacional de Ciencias Naturales, MNCN-CSIC, José Gutiérrez Abascal, 2, Madrid, 28006 Spain; CCMAR, Campus de Gambelas - Universidade do Algarve, Faro, 8005-139 Portugal; Smithsonian Tropical Research Institute, Apartado, 0843-03092 Panama; New address: Department of Botany and Plant Pathology, Oregon State University, Corvallis, OR 97331-2902 USA; BIOGES, Campus de Tafira s/n, University of Las Palmas de Gran Canaria, Las Palmas de Gran Canaria, E-35017 Canary Islands Spain

**Keywords:** Microsatellite characterization, Genetic diversity, Multiple paternity, Historical demography, Canary Islands

## Abstract

**Background:**

The giant lizard of La Gomera (*Gallotia bravoana*), is an endemic lacertid of this Canary Island that lives confined to a very restricted area of occupancy in a steep cliff, and is catalogued as Critically Endangered by IUCN. We present the first population genetic analysis of the wild population as well as of captive-born individuals (for which paternity data are available) from a recovery center. Current genetic variability, and inferred past demographic changes were determined in order to discern the relative contribution of natural versus human-mediated effects on the observed decline in population size.

**Results:**

Genetic analyses indicate that the only known natural population of the species shows low genetic diversity and acts as a single evolutionary unit. Demographic analyses inferred a prolonged decline of the species for at least 230 generations. Depending on the assumed generation time, the onset of the decline was dated between 1200–13000 years ago. Pedigree analyses of captive individuals suggest that reproductive behavior of the giant lizard of La Gomera may include polyandry, multiple paternity and female long-term sperm retention.

**Conclusions:**

The current low genetic diversity of *G. bravoana* is the result of a long-term gradual decline. Because generation time is unknown in this lizard and estimates had large credibility intervals, it is not possible to determine the relative contribution of humans in the collapse of the population. Shorter generation times would favor a stronger influence of human pressure whereas longer generation times would favor a climate-induced origin of the decline. In any case, our analyses show that the wild population has survived for a long period of time with low levels of genetic diversity and a small effective population size. Reproductive behavior may have acted as an important inbreeding avoidance mechanism allowing the species to elude extinction. Overall, our results suggest that the species retains its adaptive potential and could restore its ancient genetic diversity under favorable conditions. Therefore, management of the giant lizard of La Gomera should concentrate efforts on enhancing population growth rates through captive breeding of the species as well as on restoring the carrying capacity of its natural habitat.

**Electronic supplementary material:**

The online version of this article (doi:10.1186/s12863-014-0121-8) contains supplementary material, which is available to authorized users.

## Background

Oceanic archipelagos are considered natural laboratories for the study of evolution [1,2]. Islands normally host a large number of endemic species that originated from the immigration of a few individuals from the continent and subsequent local evolution, adaptation, and diversification [3-5]. Yet, the extraordinary biodiversity of islands is relatively fragile. Because island endemics have evolved in an environment protected by isolation, they are particularly susceptible to ecological threats (e.g., predation by or competition with invasive species, habitat loss, and human pressure) [6,7]. Additionally, loss of genetic diversity and inbreeding depression as a result of isolation and genetic drift, may contribute to the extinction of small populations on islands [6,8,9]. However, several studies [8-12] have shown that, after severe bottlenecks, some species have been able to persist for long periods of time with depleted heterozygosity levels. Ecological factors, such as the quality of the habitat, environmental stability, the purging effect of selection, and specific life history traits (e.g., mating systems and generation lengths) could counteract the impact of declines on population genetic variation [12,13]. Thus, determining the long-term survival of an island endemic species requires disentangling the relative effects of genetic and ecological (natural or human-mediated) drivers of extinction, and their relative contribution at different temporal and spatial scales, as well as characterizing potential intrinsic species traits that could enhance or slow down extinction processes [6,8,9,14,15].

**Figure 1:**
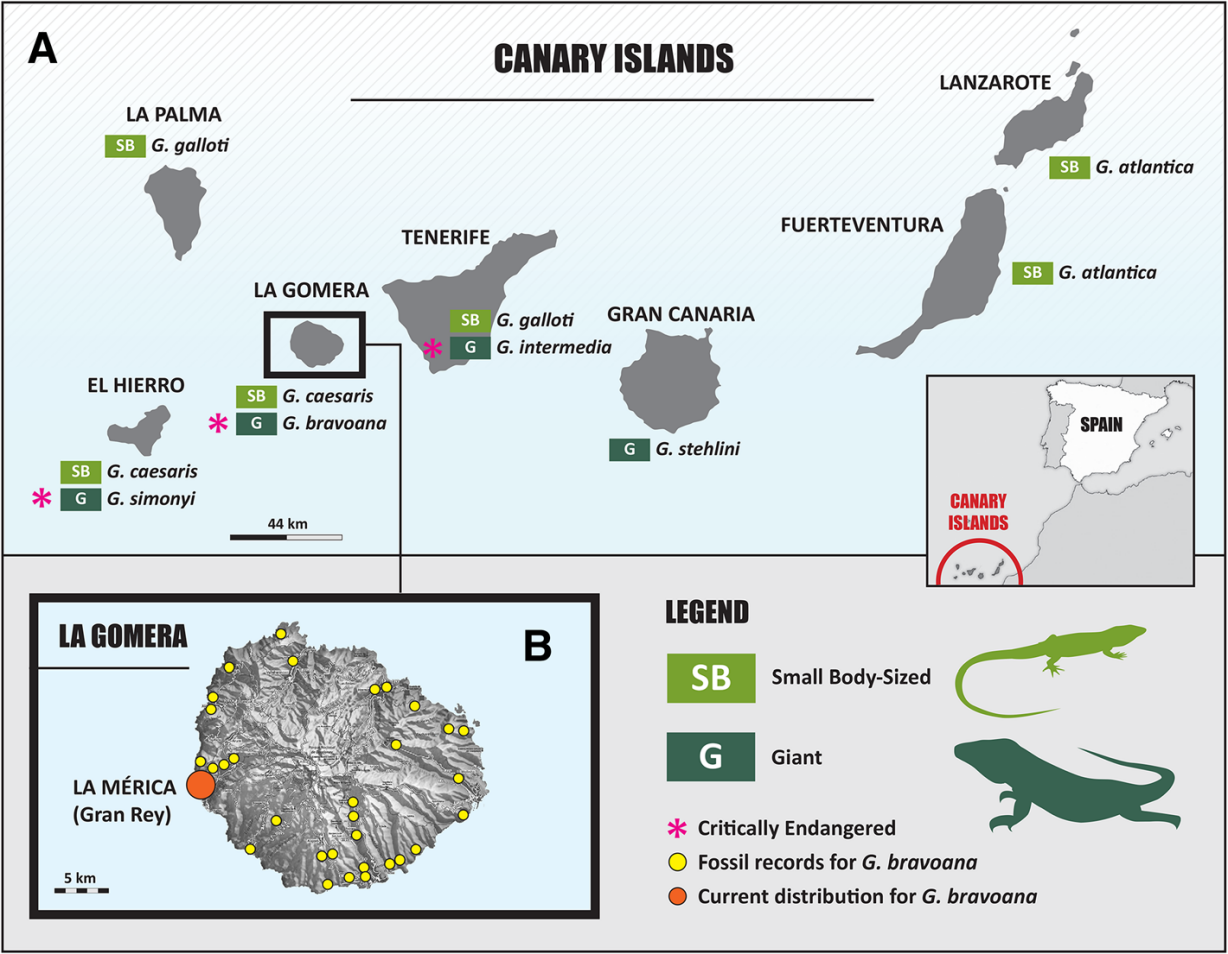
**Map of the Canary Islands showing the distribution of the** ***Gallotia*** **lizards. A)** Distribution of the small-bodied (SB) and the giant (G) lizards. The species classified as “Critically Endangered” by the IUCN (2012) are also indicated with asterisks. **B)** Topographic map of La Gomera Island showing the locality for the *G. bravoana* natural population (La Mérica cliff, near the town of Gran Rey), and the fossil record sites for *G. bravoana* [24] (indicated with yellow dots).

The genus *Gallotia* (Arnold 1973) (subfamily Gallotiinae) includes seven living lacertid species endemic to the Canary Islands that diversified upon colonization from the continent back in the early Miocene, ca. 20 million years ago (MYA) [16-18]. The sister group of *Gallotia* is the genus *Psammodromus* [19] that is found in France, the Iberian Peninsula and Maghreb. A recent phylogeny based on mitochondrial (mt) DNA sequence data [17] showed that the giant lizard from Gran Canaria*, Gallotia stehlini* is the sister group of the remaining *Gallotia* species. The next branching in the tree is between the small-bodied *Gallotia atlantica*, which inhabits eastern Canary Islands (Fuerteventura and Lanzarote), and a clade that includes all species living in western Canary Islands. This latter clade is divided into two monophyletic groups, one of small-bodied lizards, *Gallotia galloti* and *Gallotia caesaris*, and another of giant lizards, *Gallotia simonyi*, *Gallotia intermedia* and *Gallotia bravoana*. Each of these three species of giant lizards is endemic to a single island, El Hierro, Tenerife, and La Gomera, respectively. Because of their restricted distribution, these three giant lizards are highly threatened and for many years they were thought to be Extinct (Figure 1A, [16-18,20]). More recently, a phylogeny based on combined mt and nuclear sequence data recovered a very similar phylogeny that only differs in that *G. galloti* and *G. caesaris* do not form a monophyletic group because the latter species is recovered as sister group of *G. simonyi*, *G. intermedia* and *G. bravoana*, although with low statistical support [19].

Among the giant lizards, *G. bravoana*, has one of the smallest distributional ranges (Figure 1B) [21]. This species was rediscovered in 1999 (after being considered extinct since the late 19th century) when a few living specimens were found on a very inaccessible cliff called La Mérica, near the town of Gran Rey on La Gomera Island [22]. Field surveys in 2009 revealed that the whole population included ca. 160 individuals that inhabited isolated patches of <20 Km^2^ in total, restricted to La Mérica cliff [23]. Information on life-history traits of the species is still scarce, however it has been reported that individuals could live up to 60 years old, reach 40 cm of snout vent length, and produce between two to six eggs per clutch [24]. Despite active conservation efforts during the last decade, the species is still considered threatened and listed as Critically Endangered by the IUCN (http://www.iucnredlist.org/details/61502/0), and yet very little is known about the genetics and demography of its only known population.

After its rediscovery, a conservation programme (within the framework of two EU LIFE projects) was established on the island, focused mainly on captive breeding and on census monitoring of the natural population. For the captive breeding programme, nine founders (five females and four males) were captured in the wild between 1999 and 2000, and used to found the captive population. The founders reproduced successfully for the first time in 2001 at the Recovery Centre of La Gomera Giant Lizard, resulting in about 40 captive-born offspring by 2005, and 121 captive-born individuals by 2010 [23].

Evidence from fossil records and known mummified remains of more than fourteen places distributed around the island (Figure 1B) suggests that the species inhabited most of the island of La Gomera from the coast throughout the xerophilic region (except in the laurisilva subtropical forest area at high altitudes) prior to the arrival of humans (ca. 2,500 years ago) [24,25]. At that time, it is believed that lizards were very common on the island and served as a food resource for both human and domestic animals [24]. Naturalist chronicles indicate that individuals were scarce by the 15th century, suggesting that the species was already rare on La Gomera Island at the time first Europeans arrived [24]. Thus, it has been postulated that the presence of humans coupled with the human-mediated introduction of predators caused a decline in numbers of *G. bravoana* and it’s currently restricted geographical distribution. However, the possibility that decline could be the result of environmental stochastic processes such as ancient climate changes or geological (volcanic) events producing long-term fragmentation and isolation cannot be discarded [26-29]. Genetic data could allow discriminating between either alternative hypotheses by estimating whether population decline predated or not the arrival of humans to the island. Moreover, the combination of ancient natural processes and more recent anthropogenic activities may have had a synergetic effect that could best explain the current threatened status of the species.

Given the critical conservation status of the species, the study of its genetic variation was necessary to establish the best management strategy. In particular, it was important to determine whether observed reduction in population size was accompanied by depletion in levels of genetic diversity as well as to detect genetic signatures of past demographic changes (e.g., bottlenecks) and date them. Moreover, genetic data could help clarifying how historical processes (e.g., sustained population isolation and genetic drift) and more recent events (e.g., human pressure), coupled with the effect of life-history traits (e.g., mating behavior), contributed to the evolutionary history of the species.

Here, we analyze microsatellite data of *G. bravoana* for a total of 99 individuals (covering more than half of the total wild population and all 2001–2005 captive-born individuals) to estimate the overall amount of genetic variability of the species, and the allele frequency distribution between wild and captive individuals. Different coalescence-based methods were applied to examine major population demographic changes and to estimate their timing. In addition, we combined information on pedigree and genetic data of captive animals from the breeding program to perform paternity analyses and gain insights on the mating system of the species. Altogether, results presented here provide the genetic background needed for understanding the recent evolutionary history of *G. bravoana* and for implementing successful management and conservation plans for the species.

## Results

### Microsatellite variation

Eight (*GBR9*, *11*, *16*, *20*, *24*, *26*, *29* and *30*) out of the eleven loci developed in this study were polymorphic in *G. bravoana* (Table 1A), and seven of these (except *GBR26*) were also polymorphic in related species (Additional file 1: Table S1). Interestingly, locus *GBR5* was polymorphic in related species but not in *G. bravoana* (Additional file 1: Table S1). Furthermore, microsatellites *GBR11* and *16* were monomorphic in wild samples. Allele frequency homogeneity tests indicated that the probability of detecting population structure with the eight polymorphic microsatellites was relatively high (the overall power estimate from all runs was 0.714 and 0.628 for the chi-square and Fisher exact tests, respectively), and statistically significant (data not shown). When *F*_ST_ was set to zero (simulating no divergence among samples), the proportion of false significances (α error of type I) was in all cases lower than the intended value of 5%. Only one (*GBR9 versus 24*) out of the possible 28 linkage comparisons was significant (*p* <0.05, results not shown), and therefore all loci were consequently regarded as independent from each other. The majority of loci showed an overall departure from HWE due to significant heterozygote deficiency when all 99 samples were analyzed together (Table 1A). The number of alleles for polymorphic loci varied from two to seven (mean *N*_*A*_ = 3.6) when all samples were analyzed together (Table 1A). Distribution of alleles found for each locus in the wild and captive populations was very similar (Additional file 1: Figure S1).

**Table 1:** **Summary data of microsatellites La Gomera Giant lizard (** ***Gallotia bravoana*** **) for all loci (A) for wild individuals (B) and for captive samples (C)**

**A) Loci**		**Locus**	**Repeat motif**	**Tª (ºC)**	**Primers sequence 5′-3′**	**Allele range**	***N***	***N***_***A***_	***N***_***AR***_	***H***_***O***_	***H***_***E***_	***F***_***IS***_^***1***^	**GenBank No.**
	GBR5		(CA)_10_	60	F: ATATTCATCCTCCCCGCACA	177	90	1	-	-	-	-	JX661253
					F: GCATTGCGGTGAAAAAGG								
	GBR9		(GT)_17_	60	F: TGGAGGCTTCTCTTGAGGCAAGA	138-160	98	4	3.9	0.143	0.153	0.066*	JX661254
					F: CCCCCTGCCTTATGAGTTTCG								
	GBR11		(GTCT)_6_ (ATCT)_12_	60	F: CTTAACCGTCTGGTTTGCATTA	196-215	96	2	2.0	0.000	0.021	1.000*	JX661255
					F: ACTGCACCCCATAGTTGTCTTT								
	GBR15		(CA)_27_	60	F: ACTGGGGCTCAGTCTTTGTTT	140	91	1	-	-	-	-	JX661256
					F: GCGTGTCTTGTGTATATGGAATC								
	GBR16		(CA)_14_	60	F: GCAGATTTAATGGAACCTGGAG	224-238	91	2	2.0	0.011	0.033	0.664*	JX661257
					F: CAACAAAATGTGGAGTTTTAGCC								
	GBR20		(GT)_16_ (GTAA)_7_	60	F: CCACAACAAAACAAATGCAA	190-217	97	5	4.9	0.051	0.081	0.362*	JX661258
					F: GTCAGATCGACCCTCTCAGC								
	GBR24		(CA)_28_	60	F: ACTTGCAGACTATTTTGGGTT	129-167	96	4	3.9	0.531	0.512	-0.039*	JX661259
					F: ACTCGCATCCTTCTGTTACAA								
	GBR26		(CT)_15_ (CA)_13_	60	F: TGGCCACACGAGATTATTCA	103-164	90	7	7.0	0.100	0.139	0.282*	JX661260
					F: ATATCGGGCCHTTTCACA								
	GBR28		CA)_26_	60	F: ACAACACGCCTCAGTTCACA	195	89	1	-	-	-	-	JX661261
					F: GCTGCCTTGAGTGAGTCTCC								
	GBR29		(GT)_110_	60	F: GGCGTGCTTGTGTATAGGAA	132-174	97	3	2.9	0.010	0.031	0.666*	JX661262
					F: CCCAGCAGGGTTGCTTAG								
	GBR30		(CA)_13_	60	F: CGCACACTTATCCTGTCGTG	198-206	98	2	2.0	0.010	0.030	0.664*	JX661263
					F: GACAGTGAGTCATGTGTGCATTT								
	Mean (all indiv.)^2^						84.9	3.6	3.6	0.107	0.125	0.142*	
**B) Wild individuals**	GBR9						56	3	2.9	0.180	0.179	-0.90	
	GBR20						56	2	2	0.053	0.052	-0.019	
	GBR24						54	2	2	0.494	0.490	-0.127	
	GBR26						53	4	4	0.074	0.073	-0.017	
	GBR29						59	2	1.9	0.036	0.036	1.000*	
	GBR30						56	2	1.9	0.018	0.018	0.000	
	Mean (all wild indv.)						55,7	2.5	3.1	0.143	0.141	-0.052	
**C) Captive samples**	GBR9						42.0	3	2.98	0.114	0.115	0.382	
	GBR11						41.0	2	1.99	0.048	0.048	1.000	
	GBR16						36.0	2	2.00	0.080	0.081	0.660	
	GBR20						41.0	5	4.62	0.117	0.119	0.592	
	GBR24						42.0	4	3.71	0.475	0.481	-0.040	
	GBR26						37.0	7	6.92	0.226	0.229	0.413	
	GBR29						42.0	2	1.86	0.023	0.024	0.000	
	GBR30						42.0	2	1.98	0.046	0.047	1.000*	
	Mean (all captive indv.)						40.4	3.4	3.26	0.141	0.143	0.511	

*N*= number of individuals assayed. *N*_*A*_= number of alleles per locus. *N*_*AR*_=allelic richness standardized to the smallest sample size using the rarefaction method of FSTAT 2.9.3 [73]. expected (*H*_*E*_) and observed (*H*_*O*_) heterozygosities. *F*_*IS*_ = Wright’s statistics.

^1^Bold *F*_*IS*_ values are significant probability estimates after q-value correction (*p<0.05).

^2^The mean values were calculated only with the polymorphic loci data.

### Genetic diversity and population structure of the wild population

**Figure 2:**
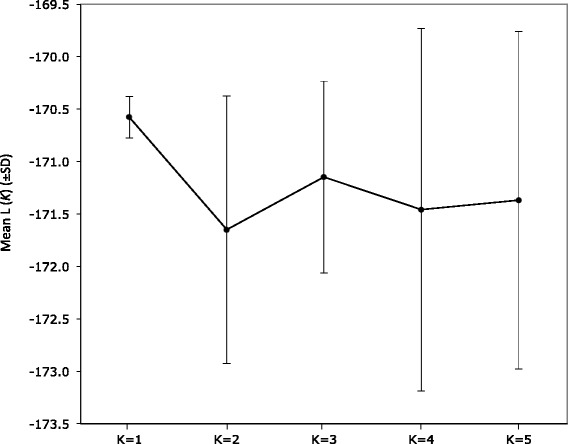
**Number of** ***Gallotia bravoana*** **populations with the highest posterior probability expressed as the mean likelihood (log P (** ***X*** **∣** ***K*** **)).**

The amount of genetic variability of the wild population was very low (mean observed heterozygosity, *H*_*O*_ = 0.143; Table 1B). Yet, the mean value of the coefficient of inbreeding was not significantly different from zero (*F*_*IS*_ = −0.05; Table 1B). Pairwise relatedness between individuals of the wild population was inferred using allele frequencies at microsatellite loci and the QuellerGT relatedness estimator (which performed best given the population composition and allele frequency distribution according to simulation analyses; Additional file 1: Figure S2). Relatedness among members (*N* = 57) of the wild population was not significantly different from zero (*r* = −0.039 ± 0.024). The number of wild populations (and the assignment of individuals to each population) was estimated using Bayesian inferences. Our results indicate that in all cases the highest posterior probability value was found at *K* = 1 and that for values of K > 1*,* every individual’s posterior assignment probability was equally split among all the specified clusters (Figure 2); hence no population structure was detected among the wild lizard samples.

### Demographic history of the wild population

The Wilcoxon test failed to detect recent bottlenecks under any kind of mutation model (IAM, TPM and SMM) of microsatellite evolution (*P* = 0.156, *P* = 0.156 and *P* = 0.109, respectively). Moreover, the allele frequency distribution obtained from the mode-shift indicator test followed a normal L-shape, indicating a larger proportion of low frequency allele classes in *G. bravoana*, and thus also supporting the absence of a recent genetic bottleneck.

**Figure 3:**
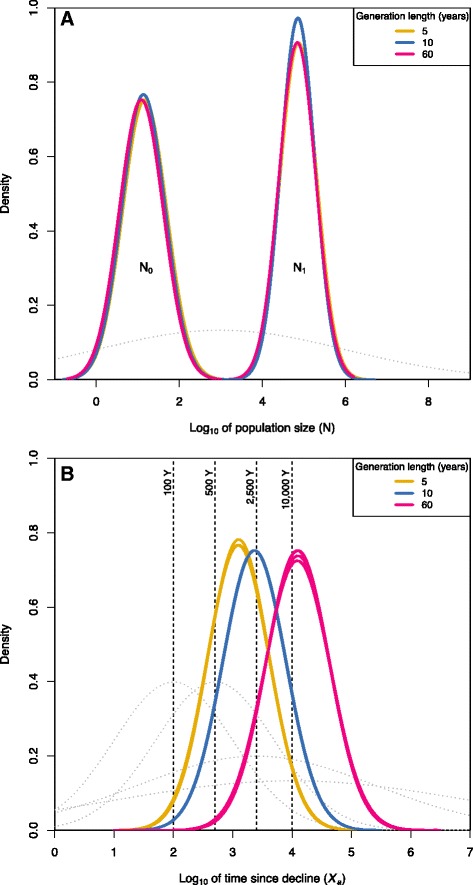
**Past demographic changes in** ***G. bravoana*** **wild populations inferred using a Bayesian coalescent approach.** Marginal posterior density of **A)** present (*N*_*0*_) and past (*N*_*1*_) effective population size represented on a log10 scale. The colors of posterior densities represent three different assumed generation times in years for the prior set analyzed, which is represented by a gray dotted line. **B)** Posterior distribution represented on a log10 scale of the time (in years) since the *G. bravoana* population decline (*Xa*), calculated using MSVAR v1.3, for the four prior sets analyzed. The colors of posterior densities represent the three different assumed generation times in years for the four prior sets analyzed, which are represented by gray dotted lines. The black vertical dashed line represents the four time periods tested (from left to right): 100; 500; 2,500 and 10,000 years (Y).

Results from the coalescent-based method applied to infer past demographic changes supported a long-term decline in *G. bravoana* (Figure 3; Table 2). The estimates of the different demographic parameters were similar irrespective of the four periods of time analyzed (Table 2). We observed that the current mean effective population size (*N*_*0*_) was always smaller than the ancestral effective mean population size (*N*_*1*_) (Figure 3A, Table 2), regardless the three values of generation time (*g*) analyzed. The mean values of *N*_*1*_ and *N*_*0*_ were 70,794 and 13, respectively. This corresponds to a reduction in effective population size (*N*_*0*_*/N*_*1*_) of around 5,400 times and that only 0.02% of the original effective population survives at present. The decline was estimated to have occurred around 221–246 generations before present, and the time estimation of the onset of the decline varies depending on the generation time prior but not on the four time periods analyzed. For a *g* = 5 years, the decline was inferred to have started around 1,230 years ago (with a confidence credibility interval of 110 - 12,023); for a *g* = 10 years the start of the decline was estimated to be around 2,344 years ago (195 - 23,442); and for *g* = 60 years the decline would have started around 13303 years ago (1,000 - 128,825) (Figure 3B, Table 2).

**Table 2:** **Full Log10 posterior estimates (and high posterior densities, HPD) of natural parameters obtained with MsVar for three different putative generation times and for four historical events that may have affected** ***G. bravoana*** **demography (see text)**

	**100 years**			**500 years**			**2500 years**			**10000 years**		
		**95% HPD**			**95% HPD**			**95% HPD**			**95% HPD**	
	**Mean**	**Lower**	**Upper**	**Mean**	**Lower**	**Upper**	**Mean**	**Lower**	**Upper**	**Mean**	**Lower**	**Upper**
**G = 5 years**												
Ancient effective population size (*N*_*1*_)	4.86	3.97	5.74	4.86	3.97	5.74	4.87	3.98	5.75	4.86	3.98	5.75
Current effective population size (*N*_*0*_)	1.17	0.08	2.17	1.16	0.05	2.16	1.17	0.07	2.16	1.16	0.06	2.17
Time since effective population size change (*X*_*a*_)	3.10	2.04	4.07	3.09	2.05	4.08	3.10	2.05	4.08	3.09	2.03	4.07
Mutation rate (*μ*)	-3.17	-3.65	-2.69	-3.17	-3.65	-2.69	-3.17	-3.65	-2.69	-3.17	-3.65	-2.69
**G = 10years**												
Ancient effective population size (*N*_*1*_)	4.85	3.97	5.74	4.86	3.99	5.76	4.86	3.98	5.74	4.86	3.97	5.75
Current effective population size (*N*_*0*_)	1.14	0.01	2.16	1.14	0.02	2.16	1.13	0.02	2.14	1.14	9E 04	2.16
Time since effective population size change (*X*_*a*_)	3.37	2.29	4.37	3.37	2.29	4.37	3.36	2.29	4.36	3.37	2.28	4.38
Mutation rate (*μ*)	-3.17	-3.65	-2.69	-3.17	-3.65	-2.69	-3.17	-3.65	-2.68	-3.17	-3.65	-2.69
**G = 60years**												
Ancient effective population size (*N*_*1*_)	4.84	3.96	5.72	4.84	3.98	5.73	4.84	3.96	5.72	4.84	3.96	5.73
Current effective population size (*N*_*0*_)	1.09	-0.04	2.13	1.08	-0.06	2.13	1.09	-0.08	2.13	1.09	-0.06	2.13
Time since effective population size change (*Xa*)	4.10	3.00	5.11	4.09	2.99	5.10	4.10	2.99	5.12	4.09	2.99	5.11
Mutation rate (*μ*)	-3.16	-3.64	-2.68	-3.16	-3.64	-2.68	-3.16	-3.64	-2.68	-3.16	-3.63	-2.67

### Multiple paternity in the captive population

The levels of genetic diversity of the captive population were low and similar to those found in the wild population (Table 1C). Overall relatedness among individuals (*N* = 20) of the captive population was low (*r* = 0.037 ± 0.056). Within the eleven clutches that hatched in captivity between 2001–2005, eight yielded a unique (monogamous) possible paternal genotype (Table 3). Fisher’s exact tests were non-significant, confirming that a single male sired the clutches (Table 3). The remaining three clutches resulted from the combination of more than one (polyandrous) male (Table 3). Multiple paternity cases never involved more than two males. Interestingly, in genetically monogamous pairings, a relatively high number of parental mismatches were detected i.e., in five cases the assigned male did not correspond with the putative father. In two out of these five cases, the obtained genotype coincided with that of the male of the previous year’s crossing, in another two cases the genotype was of one of the founder males not involved in the breeding experiment, and in another case the proposed genotype did not match any of the males used for breeding (Table 3).

**Table 3:** **Summary of breeding pairs and mating system for the** ***G. bravoana*** **individuals used for the paternity analyses**

**Year of birth**	**Female parent** ^**1**^	**Male parent**	**Nº of indiv. hatched**	**Offprings analyzed**	**Mating system**	***F***_***IS***_^**2**^
2002	*GBR2*	*GBR24*	7	*GBR64, GBR65, GBR66*	?	0.558
2003	*GBR*	*GBR22*	2	*GBR67, GBR68*	P	-1.000
2003	*GBR1*	*GBR24*	3	*GBR74, GBR75*	M	0.35
2003	*GBR8*	*GBR22*	4	*GBR69, GBR70, GBR71, GBR72*	P	-0.143
2003	*GBR16*	*GBR25*	1	*GBR73*	M	—
2004	*GBR11*	*GBR25*	3	*GBR78,GBR79*	M	0.400
2004	*GBR8*	*GBR22*	7	*GBR77*	P	-0.200
2005	*GBR2*	*GBR25*	1	*GBR80*	M	—
2005	*GBR50*	*GBR18*	8	*GBR93, GBR94, GBR95, GBR98*	M	-0.124
2005	*GBR11*	*GBR32*	7	*GBR87, GBR90, GBR92*	M	-0.500
2005	*GBR8*	*GBR26*	5	*GBR81, GBR82, GBR83, GBR84, GBR85*	M	-0.600

M: monogamous, P: polygynous, ?: unknown. *F*_*IS*_ = Wright’s statistics.

^1^The code name correspond to the studbook ID # in Additional file 1: Table S2.

^2^None of the *F*_*IS*_ value were significant.

## Discussion

We analyzed for the first time the genetic structure and demographic history of a highly threatened Canary Island giant lizard [22], *G. bravoana*, which shows an extremely reduced population number (ca. 160 individuals in the wild) and a severely reduced geographical distribution (<20 Km^2^) in La Gomera [24]. Some of the methodological limitations related to the natural small population size were overcome by maximizing sampling effort in order to cover more than half of the wild population diversity of the species, and by using powerful statistical tools based on coalescence.

Overall, the eight polymorphic species-specific microsatellite loci used in this study showed no significant linkage disequilibrium, but otherwise very low levels of genetic diversity. The observed overall departure from HWE could be explained in terms of admixture of genetically distinct cohorts (Whalund effect) given that the pattern of HWE departures changed completely when only the samples from the wild were analysed (only the *F*_*IS*_ of *GBR29* was significant, Table 1B). Levels of heterozygosity in the wild and captive populations were similar indicating that the captive population could be considered a sound representation of the genetic variability found in the wild. Heterozygosity values herein reported are lower than those previously estimated for *G. bravoana* based on different microsatellite loci (mean *H*_*O*_ = 0.42, [30]) and those reported for other species within the genus (*G. atlantica*, mean *H*_*O*_ = 0.86; [31], and *G. galloti*, mean *H*_*O*_ = 0.79; [32]) that are considered as Least Concern. Moreover, the values are also lower to those reported for island squamate species described as Vulnerable (e.g., the Komodo Dragon, [33]) and Endangered (e.g., the Balearic Island Lilford’s Wall Lizard, [34]). They are also lower to the values reported for Critically Endangered species such as e.g., the Reunion Cuckoo shrike [9]). Therefore, although direct comparison of heterozygosity levels between different microsatellite loci is difficult [35], the detected low values for *G. bravoana* seem to reinforce its genetically depleted status.

Despite long-term isolation of the population and low genetic variability values, the overall estimates of relatedness indicated low inbreeding within *G. bravoana*. The *r* values appear to be comparable to those reported for social lizards [36,37]. However, given the large values of variance obtained, interpretation of the results should be taken cautiously, and a larger number of individuals need to be included in further analyses.

We failed to detect any population structure based on the Bayesian clustering analysis, which suggests that individuals intermix freely in the single population of La Mérica cliff. In fact, we observed that the wild population was in HWE suggesting random mating and gene flow between individuals. Altogether, results indicate that *G. bravoana* is capable of actively dispersing across the different altitudinal patches despite the orographic difficulties of the steep terrain of La Mérica cliff.

The effect of human pressure on island biodiversity has been well documented and is considered one of the main causes of population declines and extinctions of many endemic island species [8,38,39], and in particular of several giant squamates such as the giant skink (*Chioninia coctei*) from Cape Verde archipelago [40], the Round Island burrowing boa (*Bolyeria multocarinata*) [41], the giant Jamaican galliwasp (*Celestus occiduus*) [42], and the Martinique giant Ameiva (*Ameiva major*) [42]. However, human pressure and associated deterministic factors are likely not the exclusive cause of the decline of island endemic populations, and there is a long-standing debate on the relative contribution of stochastic events of environmental and genetic nature in extinction [9,14]. While climatic changes likely caused population decline in fruit bats (*Cynopterus brachyotis*) of the Indomalayan region about 30,000 to 58,000 years ago [43] and in the Copaia tree (*Jacaranda copaia*) in the Panama region about 16,000 to 19,000 years ago [44], the effect of human pressure is likely behind the collapse of mouse lemurs of the genus *Microcebus* from Madagascar 500 years ago [35], and of orang-utans from North Eastern Borneo 200 years ago [38].

In the case of the giant lizard of La Gomera, the results of classic equilibrium-based methods to test for bottlenecks discard a recent human-mediated population collapse in the last 700–1,400 years (corresponding with the time frame limits of the Bottleneck test of 2Ne – 4Ne generations [45]). The results of the coalescent analysis showed a long-term decline and estimated a strong reduction to a current effective population size of 13, what is congruent with the present day effective size of the population (as estimated through census monitoring campaigns). Although the different coalescent analyses agreed on the number of generations since the decline of the population (around 230), dating the onset of the decline was more difficult and strongly dependent on what generation time was used as a prior. The longest generation time prior favored the hypothesis of a continuous decline of *G. bravoana* populations since at least 13,000 years ago, which could be related to environmental disturbances such as past climatic changes or volcanic eruptions. However, shorter generation time priors supported instead that the onset of the decline would be related to the human arrival to the islands about 2,500 years ago. In fact, the 95% high posterior densities associated to the estimates were relatively large and thus, it is not possible to fully discriminate among competing scenarios, as well as to discard a synergetic effect of human activities and lon-term environmental or genetic factors in the decline of the giant lizard populations on the island.

The Canary Islands giant lizards are characterized by their larger body size, longer life span, and lower reproductive rates compared to small-bodied lizards. These are all life-history traits that contribute to genetic drift in small populations and eventually may lead to extinction. For instance, the longer generation times of the giant lizards would contribute to the overlapping of generations, and following the Moran model [46] for genetic drift, this would accelerate the genetic drift process in small populations. It would also contribute to a reduction in the fixation of mutations that could lead to higher fitness and adaptation and as well as a reduction in genetic diversity, the effective population size and allele frequencies. Deleterious mutations under inbreeding could become fixed to a load untenable for the population and lead to extinction such as in the case of the giant skink of Cape Verde [40]. However, genetic drift is stochastic in nature, and the process does not necessarily need to end in extinction, as is the case of the giant lizard of La Gomera. The combined input of both genetic and ecological factors on population viability may explain long-term persistence of *G. bravoana* despite low genetic diversity.

Although it is widely accepted that substantial genetic variation is necessary for the long-term survival of species [11,12,47], evidence is accumulating for the capacity of many species (e.g., the Raso lark [10], the Reunion cuckoo shrike [9], the Madagascar fish-eagle, [48], or the Amsterdam albatross [49]; within squamates, the Gran Cayman blue iguana, *Cyclura lewisi* [50], could represent a similar case to *G. bravoana*,) to pass through historical bottlenecks and persist with small population sizes and low genetic diversity. Moreover, it has been shown that minimal management and conservation actions for these threatened species were enough to enhance population growth rates [12], and bring them back from the extinction vortex. In this regard, it is important to note that low genetic variation as inferred form neutral loci such as microsatellites does not predict the variability and evolutionary potential of loci under selection (and thus ecologically important) [11,12]. Selection can act retaining genetic variation important for adaptation and purging genetic load after a bottleneck [12,51].

From the genotype comparison analysis on the captive population, we observed a frequency of multiple paternities (three out of the eleven clutches examined had multiple sires, 27%) of similar level to those reported in other social lizard communities, such as *Egernia whitii* (11.6%, [52]), *Egernia stokesii* (25%, [53]) or *Egernia saxatilis* (20%, [54]). Moreover, parental mismatches were detected among hatches derived from monogamous pairs. Since a single female and male pair was put together during a short period of mating confinement, parental mismatches could be explained by the existence of previous or posterior crossings of the female with other males within the breeding season. Also, the parental mismatches could be the result of inclusion of females previously inseminated in the field in the breeding program. Overall, our results strongly support that *G. braovana* females may be able to retain sperm. Although rare in birds and mammals, long-term sperm storage (i.e., for months or even years) in natural populations are more common in reptiles and amphibians (e.g. *Crotalus adamanteus*, [55], *Alligator missippiensis*, [56], *Desmognathus ocoee*, [57], but see [58] and references therein). Therefore, of the possible explanations for the observed paternity pattern, multiple matings by the female with different males, combined with the ability for long-term sperm retention is the most likely.

It is plausible that multiple mating between *G. gallotia* individuals may be used as an indirect strategy to avoid inbreeding [37,59,60], which could potentially benefit population viability. The overall low inbreeding values obtained are in accordance with this expectation, although the associated values of variance were high, and further analyses with larger samples are needed to confirm this hypothesis. Other mechanisms to avoid inbreeding have been described in lizards (e.g., kin recognition and sex-bias dispersal, [36,37,49,61,62]); however, determining the influence of each of these mating strategies on *G. bravoana* requires data not yet collected for this species. It is not clear whether or not the pattern of multiple paternities observed in the captive population may be representative of mating in the wild population since mating in captivity did not happen in a random way. However, the confinement of the wild population to a single cliff, the reduced number of breeding individuals, and the existence of a single evolutionary unit seem to provide the conditions that could favor the multiple paternity behavior also in the wild (see [63] for an example of multiple paternity reported in natural populations of lacertid lizard species).

## Conclusions

The demographic history analyses performed in this study indicate that the original *G. bravoana* population was made up of thousands of individuals that suffered a long-term gradual demographic decline that was estimated to have started between 1,200–13,000 years ago (depending on the assumed generation time and with large confidence intervals). Fossil records suggest that its decline was accompanied by a continuous reduction in geographical distribution, resulting in its present-day restricted distribution on La Mérica cliff. Ever since, the only known population has survived isolated, with a low effective number and low levels of genetic diversity. Wild individuals of *G. bravoana* act as a single evolutionary unit, as supported by the Bayesian clustering results, and gene flow is unrestricted throughout La Mérica cliff. Relatedness analyses indicate low levels of inbreeding (although these results should be taken cautiously as they present large values of variance and thus a larger number of individuals and loci need to be included in further analyses). Kinship analyses of the captive population support *G. bravoana* monogamous and polygynous pairings, presumably conducted through multiple mating and long-term sperm retention mechanisms, which might have contributed to avoid inbreeding and towards species persistence. This suggests that the deleterious genetic load associated to the gradual decline suffered by the species may have been purged in the population, which therefore retains the (adaptive) potential to recover if population growth rates are enhanced through the breeding program.

## Methods

### Sample collection and DNA extraction

To collect samples from wild individuals, several capture/recapture campaigns were performed throughout La Mérica cliff (subdivided into several ledges or promontories of climb-sampling due to its isolation and inaccessibility). From 2001 to 2005, we sampled a total of 57 specimens (Additional file 1: Table S2). Given that the 2005 census estimated 100 individuals in La Mérica [64], we assume to have captured more than half of the population. Blood samples were taken from adults that were captured and subsequently released into the wild, whereas tissue samples (skin or muscle) were taken from dead individuals (Additional file 1: Table S2). All samples were collected with the appropriate permissions issued by the Government of Canarias under the service agreement no. 03103 corresponding to the European Life Project n° LIFE 02 NAT-E-008614.

From 2001 to 2005, a total of 42 juveniles born in captivity were analyzed. Crosses of five females and four males captured in the wild produced 27 juveniles during the five years (Additional file 1: Table S2). In addition, in 2005, two males (*GBR18* and *32*), born in captivity in 2001, were included in the breeding program and contributed with 15 individuals (Additional file 1: Table S2). Crossing experiments were as follows. All females and males were kept together, until specific couples were separated for designed crosses. After crosses, individuals were returned to common facilities. Crosses were designed so that all males and females would breed over the years, but no genetic information was taken into account. Clutches were incubated in different containers, and 0.5 ml blood sample was obtained from juveniles after hatchling. Sex was determined via visual examination of the sexual characters in adults and subadults (such as the relative size of the head and the presence/absence of a hemipenis). Sex of juveniles was determined once they attain sexual maturity.

All samples were stored in ethanol at −20°C and total DNA was extracted using the QIAGEN DNeasy tissue kit (Qiagen) following manufacturer’s conditions.

### Microsatellite characterization

Specific microsatellite markers were isolated and developed for *G. bravoana* (Table 1). An enriched partial genomic library was generated from DNA of an individual captured in the wild (studbook ID #*GBR1*) as previously described [65,66] using a method that relies on the construction of a genomic library of blunt ended DNA fragments enriched for GT repeat sequences ligated to SNX linkers [67-69]. A total of 80 positive clones were sequenced, and 18 that contained simple GT dinucleotide repeats were selected for primer design using PRIMER3 software [70]. From those, 11 microsatellites (GenBank accession numbers JX661253-JX661263) were successfully amplified after PCR optimization, and were subsequently used to genotype the 99 samples. PCR amplifications consisted of one cycle of denaturing at 95°C for 2 min; 30 cycles of denaturing at 94°C for 30 s, annealing for 30 s at 52°C - 60°C, and extension at 72°C for 90 s; followed by one cycle of 15 min extension at 72°C. Reactions contained approximately 10 ng of sample DNA, 0.5 U of Taq DNA polymerase (Eppendorf), 0.4 μM of each primer, 0.2 mM of each dNTP, 2–3 mM MgCl_2_, 1×Taq Buffer Advanced (Eppendorf, 20 mM Tris–HCl, pH 8, 100 mM KCl, 0.1 EDTA, 1 mM DTT), and DEPC-water to a final volume of 15 μl. Forward primers were labeled with fluorescent dyes, and amplified PCR products were run on an ABI Prism 3730 DNA Analyzer (using the GeneScan™-500 LIZ® Size Standard, Applied Biosystems). Allele scoring was performed using GeneMapper v3.7 (Applied Biosystems). Approximately 35% of the samples were re-run to assess repeatability in scoring. Moreover, polymorphic primers were cross-amplified in seven species of the subfamily Gallotiinae (*G. intermedia, G. simonyi, G. caesaris, G. atlantica, G. stehlini, G. galloti* and *Psammodromus algirus*) to test the amplification range and polymorphism of the specific primers in closely related species (Additional file 1: Table S1).

### Genetic diversity analyses

The observed (*H*_*O*_) and expected (*H*_*E*_) heterozygosities [71], number of alleles (*N*_*A*_) and the number of alleles standardized for the smallest sample size were calculated using GENEPOP v 4.0 [72] and FSTAT [73] programs. Heterozygote deficiency according to departures from Hardy-Weinberg equilibrium (HWE), Wright’s *F*_*IS*_ statistic estimations, and linkage disequilibrium were determined using Markov chain Monte Carlo (MCMC) runs for 1,000 batches, each of 2,000 iterations, with the first 500 iterations discarded before sampling [74]. Correction for multiple testing (type I error) was performed using the false discovery rate (FDR) approach [75] using the R package QVALUE [76]. Wild and captive samples were analyzed both independently and combined into a single data set.

The program POWSIM [77] was used to estimate the statistical power to detect significant genetic differentiation with the newly developed microsatellite markers. All individuals were used for testing allele frequency homogeneity at each of the polymorphic loci separately, or combined with Fisher’s exact and traditional chi-squared tests. Burn-in consisted of 1,000 steps followed by 100 batches of 1,000 steps.

### Population structure analyses

To determine possible population differentiation in the wild lizard population, a Bayesian clustering approach (as implemented in STRUCTURE [78]) was used. The number of populations (*K*) with the best-estimated probability (lnProb (D)) value was calculated assuming an admixed model and a uniform prior probability of *K* [78]. We performed a series of independent runs for *K* from one to five putative populations. MCMC consisted of 5 × 10^6^ burn-in iterations followed by 5 × 10^5^ sampled iterations.

### Relatedness analyses

Pairwise coefficients of relatedness (*r*) among adult lizards from the wild population and among captive-born individuals of 2005 were estimated using COANCESTRY [79]. We chose four commonly-used moment relatedness estimators (see [80] for a comparison of their performance): the regression based estimator (QuellerGT, [81]), the regression based method-of-moments estimator (LynchRd, [82]), Wang’s estimator (Wang, [83]), and the Triadic Likelihood estimator (TrioML, [80]). To determine which of these estimators perform best with our data, we generated three simulated genotype data sets based on observed allele frequencies of the wild *G. bravoana* population and three types of True Relatedness relationships (unrelated siblings, UR, were *r* = 0.0; half-siblings, HS, were *r* =0.25; and full siblings, FS, were *r* = 0.5). The relative performance of the four estimators was calculated based on the proportion of variance estimated by the True Relatedness and the observed (simulated) relatedness composition (following the approach of other authors: [84-86]). Monte Carlo simulations were performed using the “True Relatedness to be Simulated” option in the software to specify the above three types of relationships based on 4,000 multilocus genotypes [79]. For each estimator, the 95% confidence intervals were generated with bootstrapping (1,000 replicates across loci). The mean observed relatedness (and its estimated variance), and the theoretically expected relatedness values were compared using a two-tailed *t*-test. The normality of distribution was previously checked using the Kolmogorov-Smirnov test [87]. The coefficient *r* was calculated for all wild individuals (*N* = 57 individuals) with the estimator that performed best based on the simulation (using a bootstrapping of 10,000 replicates). Due to the overall small range in progeny size in the captive population, only the genotypes for juveniles that hatched in the most successful year (2005; four clutches of a total of *N* = 20 individuals) were used for relatedness estimations with the estimator that performed best.

### Demographic history analyses

Tests based on summary statistics as well as Bayesian approaches were applied to infer the population demography history of the wild population (*N* = 57). First, possible severe reductions in effective population size were assessed using BOTTLENECK [88,89]. This method assumes that recently bottlenecked populations should exhibit a significant excess of heterozygosity (*H*_*E*_) compared to the expected one at mutation-drift equilibrium. Analyses were carried out assuming three different mutation models: (i) infinite allele (IAM), (ii) stepwise mutation (SMM), and (iii) two-phase (TPM, with 70% stepwise, 30% variable), and applying the Wilcoxon signed rank test for statistical detection of *H*_*E*_. Estimations were based on 10,000 replicates. Also, the mode-shift test [90] contained in BOTTLENECK was performed. The assumption behind the test is that a population under mutation-drift equilibrium is expected to have a larger proportion of alleles with low frequencies, whereas a population that has undergone a recent bottleneck tends to lose rare alleles increasing the frequencies of common alleles.

Recently, coalescent-based methods [91,92] were developed for estimating the likelihood of past demographic changes from present-day samples in a more efficient way than classic equilibrium-based methods based on heterozygosity or allele frequency distribution departures [90,93,94]. Therefore, we modeled past demographic changes in *G. bravoana* wild population using a Bayesian coalescent approach as implemented in MSVAR v1.3 [43,91]. This program is able to provide multilocus posterior distribution estimates of four natural parameters under a model of exponential change in effective population size: the ancestral effective population size (*N*_*1*_), the current effective population size (*N*_*0*_) after *T*_*a*_ number of generations of expansion/decline processes, the time since the effective population size change in absolute years (*X*_*a*_ = *g* × *T*_*a*_*,* where *g* is the generation time), and the mutation rate (*μ*.) [43].

Information obtained from individuals breeding in captivity indicates that *G. bravoana* females attain sexual maturity within the first four to six years (JAM personal obs). In the wild, adults can live up to 10–18 years [24]. Additionally, skeleton chronology studies of fossil records suggest that 500 years ago adults could live up to 50–60 years [24]. Due to the wide variance in the estimate of the generation time and its potential sensitivity in the Bayesian performance, we decided to do exploratory runs using different values for this parameter (*g* = 5, 10 and 60 years) in order to place broad confidence intervals around our estimates of absolute time since the population change.

We tested four time periods or historical events that could have most affected *G. bravoana* demography (following the approach of [9,35]): i) the time of main climate oscillations that occurred at the end of the Pleistocene glaciations, ca. 10,000 years ago; ii) Time of first human settlements on the Canary islands, 2,500 years ago [25], and the likely first introduction of domestic species, (e.g., goats and cats) that possibly accompanied them [24]; iii), Time of the first European settlements and further human population growth, 500 years ago [24]; and iv) Time of intensive agriculture, use of pesticides, and further impacts due to urbanization and degradation of coastal environment, about 100 years ago [95,96].

For all analyses, we ran five independent MCMC chains. Each chain was run for 6 × 10^9^ iterations and thinned at each 5 × 10^4^ interval. Using the R package BOA [97] we assessed the convergence among five chains by multivariate potential scale reduction factor statistics [98,99]. In addition, using BOA, we estimated the mean, standard deviation, and 95% highest posterior density (HPD) for the natural parameters *N*_*0*_, *N*_*1*_, *Xa*, and *μ* using a burn-in of half of the five merged chains. The log-normal priors (means and standard deviations) and hyperpriors (means and variances for means and variances) are described in Additional file 1: Table S3.

### Multiple paternity analyses

Given that maternal genotypes were known only for captive lizards, the genotypes of their offspring were used to calculate exclusion probabilities (*P*_*E*_) and maximum likelihoods of paternal genotypes of the breeding program, using GERUD [100]. Results could render in either a unique or multiple parental genotypes. For the former, Fisher’s exact tests were performed to test whether loci conformed to the expectations of Mendelian segregation in a monogamous mating [101]. For the latter, priority scores of the different solutions were ranked by likelihood (using the “Known Mother” menu option in the software).

Given the design of the study, it is relatively easy to detect the presence of null alleles in maternal lines within the progeny array. In fact, for each litter examined, the genotype of their corresponding mother was accepted by the software, and used in the analysis (meaning that all progeny shared at least one allele at each locus with their mother). Only two offspring (studbook ID #GBR64 and GBR65, Additional file 1: Table S1) failed to match at any locus with the maternal genotype (probably due to *de novo* mutation or genotyping errors), and were excluded from the analysis. Moreover, null alleles in the paternal line may lead to incorrect assignment in cases of multiple paternities. To minimize this effect, only the offspring that were successfully amplified for all loci were included in the analysis (which led to a range from one to five per clutch). On the other hand, since all loci conformed to the expectations of HWE when the wild population was analyzed (Table 1B), we considered that null alleles did not bias our estimation of paternal contribution [101].

## Additional file

Additional file 1: Figure S1. Distribution of alleles found for each locus in the wild and captive populations. **Figure S2.** Performance comparison of different relatedness (r) estimators and null hypothesis of True Relatedness relationships **Table S1.** Cross-species amplifications of 11 specific *Gallotia bravoana* microsatellites for all the related species within Gallotiinae. **Table S2.** Samples included in the study. **Table S3.** Log-normal priors and hyperpriors used for MsVar analysis performed on *Gallotia bravoana* samples.
