# Supplementary material for: Island survivors: population genetic structure and demography of the critically endangered giant lizard of La Gomera, Gallotia bravoana
Source: BMC Genet. 2014 Nov 25;15:121. doi: 10.1186/s12863-014-0121-8 (PMC4254221; doi:10.1186/s12863-014-0121-8)
Supplement: Additional file 1: Figure S1. — Distribution of alleles found for each locus in the wild and captive populations. Figure S2. Performance comparison of different relatedness (r) estimators and null hypothesis of True Relatedness relationships Table S1. Cross-species amplifications of 11 specific Gallotia bravoana microsatellites for all the related species within Gallotiinae. Table S2. Samples included in the study. Table S3. Log-normal priors and hyperpriors used for MsVar analysis performed on Gallotia bravoana samples. [file 12863_2014_121_MOESM1_ESM.pdf]

Fig. S1. Distribution of alleles found for each locus in the wild and captive populations. The size of each dot is proportional to the allele frequency.

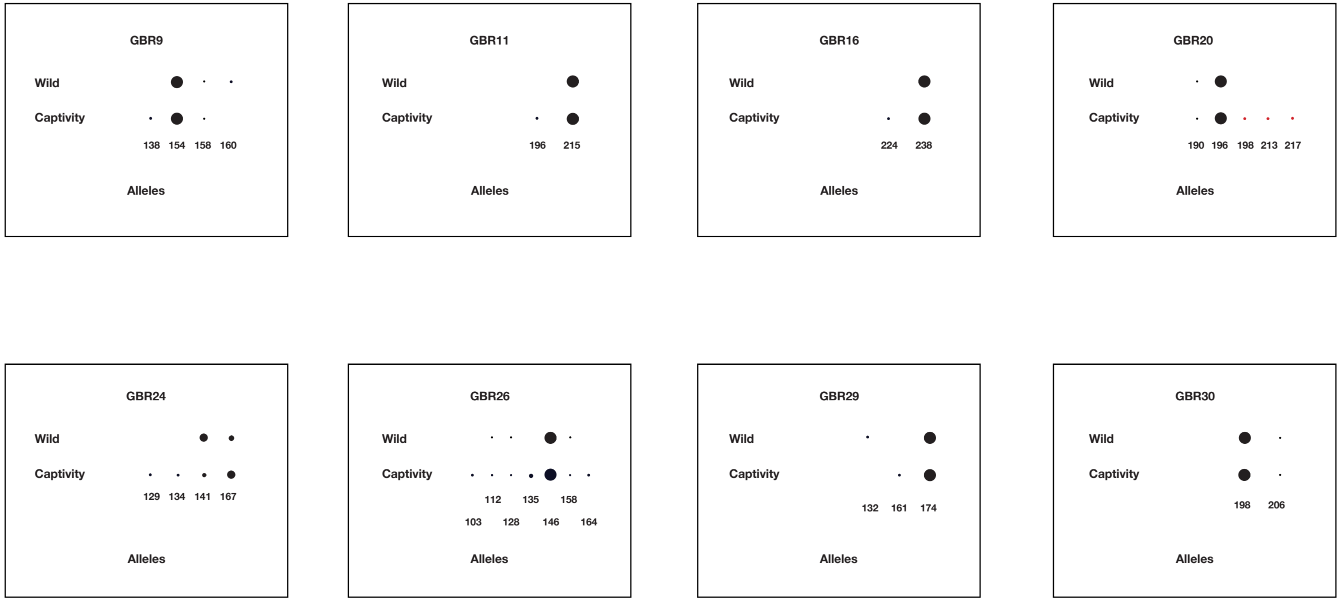

Fig. S2. Performance comparison of different relatedness (*r*) estimators and null hypothesis of True Relatedness relationships (unrelated siblings, UR, were *r* = 0.0; half-siblings, HS, were *r* = 0.25; and full siblings, FS, were *r* = 0.5): the Triadic Likelihood estimator (TrioML, [1]), the Queller estimator (QuellerGT, [2]), the Wang estimator (Wang, [3]), and the regression based method-of-moments estimator (LynchRd, [4]). Each bar indicates the mean relatedness and standard deviation (mean *r* ± SD) per relatedness estimator. Significance levels are also indicated (**P*<0.05; ** *P*<0.01; *** *P*<0.001).

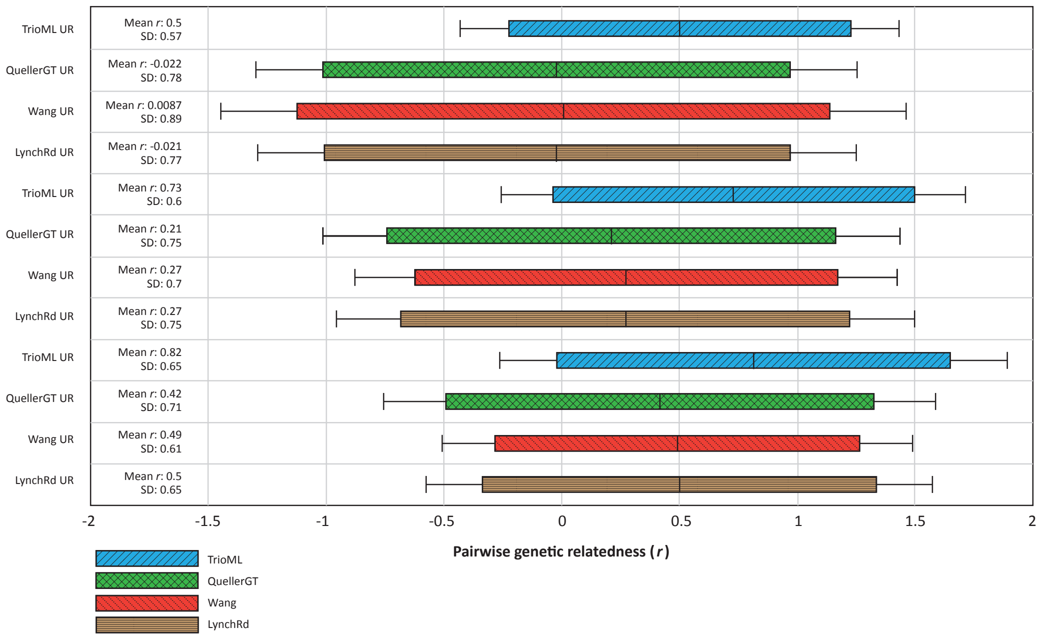

The performance of each estimator was obtained as the difference between the mean observed and expected *r* value in each True Relatedness kinship category (FS: where *r* =0.5; HS: *r* =0.25; UR: *r* =0.0). The sampling error was calculated as the standard deviation (SD) of the relatedness estimates for each dataset. Since we observed that the data for all groups deviate from a normal distribution (Shapiro-Wilks normality test, $P < 0.00$), we ran the Wilcoxon matched test (analogous to the paired *t*-test) instead.

Our results indicated that for our datasets the means of most of the relatedness estimates for the three kinship categories deviate significantly from expected relatedness values (i.e., in nine out of the 12 tests performed, Supplementary Fig. S3). The most affected was the TrioML estimator that showed the strongest significant

upward bias in all cases and an average bias across relationships of 0.51. The two estimators that showed the lower difference between observed and expected values were QuellerGT and Wang estimators (mean average bias across the three relationships was 0.072 and 0.169, respectively). However, the comparison of the SD variance between the estimates indicated that QuellerGT performed better, and was selected for further analysis.

Finally, when considering the different relationships analyzed, we observed that the population defined by a higher proportion of related individuals (FS, where $r = 0.5$) performed better, which could be indicative of the true genetic relatedness of our study population.

1. Wang J: **Triadic IBD coefficients and applications to estimating pairwise relatedness**. *Genetics Research* 2007, **89**(3):135-153.
2. Queller DC, Goodnight KF: **Estimating relatedness using genetic-markers**. *Evolution* 1989, **43**(2):258-275.
3. Wang JL: **An estimator for pairwise relatedness using molecular markers**. *Genetics* 2002, **160**(3):1203-1215.
4. Lynch M, Ritland K: **Estimation of pairwise relatedness with molecular markers**. *Genetics* 1999, **152**(4):1753-1766.

Supplementary Table S1. Summary data for cross-species amplifications of 11 specific *Gallotia bravoana* microsatellites for all the related species within Gallotiinae. The number of individuals *(N)* analyzed are also included*

Locus	*G. intermedia* (*N*=8)	*G. simonyi* (*N*=8)	*G. caesaris* (*N*=8)	*G. atlantica* (*N*=8)	*G. stehlini* (*N*=8)	*G. galloti* (*N*=8)	*P. algirus* (*N*=8)
GBR5	—	+ (2, 171-173)	—	—	± (2; 157-171)	—	—
GBR9	+ (2; 138-140)	+ (2; 149-156)	+ (2; 138-140)	± (3; 123-154)	+ (7; 140-162)	+ (3; 138-149)	+ (6; 131-170)
GBR11	+ (4; 211-242)	+ (4; 207-219)	+ (6; 175-200)	+ (5; 200-215)	+ (7; 200-232)	+ (4; 179-207)	+ (1; 145)
GBR15	—	—	—	—	—	—	—
GBR16	+ (3; 230-242)	+ (3; 219-242)	+ (1; 224)	+ (4; 207-227)	+ (9; 228-259)	+ (1; 224)	+ (1; 225)
GBR20	+ (4; 185-196)	+ (4; 185-194)	+ (8: 181-224)	+ (4; 171-187)	+ (6; 167-191)	+ (5; 191-210)	+(3; 194-201)
GBR24	+ (5; 147-159)	+ (1; 145)	+ (8; 129-163)	± (1; 153)	± (3; 143-153)	+ (3; 145-171)	± (1; 201)
GBR26	—	—	—	—	—	—	—
GBR28	—	—	—	—	—	—	—
GBR29	—	—	—	—	—	—	± (2; 194-196)
GBR30	+ (3; 170-200)	+ (1; 198)	+ (2; 198-206)	+ (4; 186-194)	+ (1; 186)	+ (4; 196-222)	+ (1; 225)

* The number of alleles and the allele range are presented in parentheses; + allele amplification success; ± low intensity allele amplification; - no allele amplification.

Table S2. Samples included in the study. The studbook identification number, the origin of sampling, date of sample collection, and the source for DNA extraction are also included.

Studbook ID	Origin of sampling	Date of sampling	DNA source
*GBR18	Vivarium	2001	Tissue
GBR19	Vivarium	2003	Tissue
*GBR32	Vivarium	2001	Tissue
GBR33	Vivarium	2001	Tissue
GBR35	Vivarium	2002	Blood
GBR64	Vivarium	2002	Tissue
GBR65	Vivarium	2002	Tissue
GBR66	Vivarium	2002	Tissue
GBR67	Vivarium	2003	Tissue
GBR68	Vivarium	2003	Tissue
GBR69	Vivarium	2003	Tissue
GBR70	Vivarium	2003	Tissue
GBR71	Vivarium	2003	Tissue
GBR72	Vivarium	2003	Tissue
GBR73	Vivarium	2003	Tissue
GBR74	Vivarium	2003	Tissue
GBR75	Vivarium	2003	Tissue
GBR76	Vivarium	2003	Tissue
GBR77	Vivarium	2004	Tissue
GBR78	Vivarium	2004	Tissue
GBR79	Vivarium	2004	Tissue
GBR80	Vivarium	2002	Tissue
GBR81	Vivarium	2005	Tissue
GBR82	Vivarium	2005	Tissue
GBR83	Vivarium	2005	Tissue
GBR84	Vivarium	2005	Tissue
GBR85	Vivarium	2005	Tissue
GBR86	Vivarium	2005	Tissue
GBR87	Vivarium	2005	Tissue
GBR88	Vivarium	2005	Tissue
GBR89	Vivarium	2005	Tissue
GBR90	Vivarium	2005	Tissue
GBR91	Vivarium	2005	Tissue
GBR92	Vivarium	2005	Tissue
GBR93	Vivarium	2005	Tissue
GBR94	Vivarium	2005	Tissue
GBR95	Vivarium	2005	Tissue
GBR96	Vivarium	2005	Tissue
GBR97	Vivarium	2005	Tissue
GBR98	Vivarium	2005	Tissue
GBR99	Vivarium	2005	Tissue
GBR100	Vivarium	2005	Tissue
GBR1	Veta Fuente	1999	Tissue
*GBR2	Quiebracanillas	2000	Blood
GBR3	Veta Fuente	2000	Tissue
GBR4	La Mérica	2000	Tissue
GBR5	Quiebracanillas	2000	Blood
GBR6	Quiebracanillas	2000	Blood
GBR7	Conejo	2000	Blood
*GBR8	Conejo	2000	Blood
GBR9	Conejo	2000	Blood
GBR10	Conejo	2000	Blood
*GBR11	Conejo	2000	Blood
GBR12	Conejo	2001	Blood
GBR13	Quiebracanillas	2001	Blood
GBR14	Conejo	2001	Blood
GBR15	Conejo	2001	Blood
*GBR16	Quiebracanillas	2001	Blood
GBR17	Veta Fuente	2001	Tissue
GBR20	La Calera	2001	Tissue
GBR21	Veta Fuente	2001	Tissue
*GBR22	Quiebracanillas	2001	Blood
GBR23	Conejo	2001	Blood
*GBR24	Veta Fuente	2000	Blood
*GBR25	Quiebracanillas	2001	Blood
*GBR26	Quiebracanillas	2001	Blood
GBR27	Quiebracanillas	2002	Blood
GBR28	La Mérica	2002	Tissue
GBR29	Cacas	2002	Blood
GBR30	Veta Fuente	2002	Blood
GBR31	Cacas	2002	Blood
GBR34	Veta Fuente	2002	Blood
GBR36	Veta Fuente	2002	Blood
GBR37	Cacas	2002	Blood
GBR38	Veta Fuente	2002	Tissue
GBR39	Veta Fuente	2003	Tissue
GBR41	Cacas	2003	Tissue
GBR42	Conejo	2003	Blood
GBR43	Conejo	2003	Blood
GBR44	Conejo	2003	Blood
GBR45	Quiebracanillas	2003	Blood
GBR46	Conejo	2003	Tissue
GBR47	Conejo	2003	Blood
GBR48	Conejo	2003	Blood
GBR49	Conejo	2003	Tissue
*GBR50	Conejo	2003	Blood
GBR51	Conejo	2003	Blood
GBR52	Conejo	2003	Blood
GBR53	Conejo	2003	Blood
GBR54	Conejo	2003	Blood
GBR55	Conejo	2003	Tissue
GBR56	La Mérica	2004	Tissue
GBR57	La Mérica	2004	Tissue
GBR58	La Mérica	2004	Tissue
GBR59	La Mérica	2004	Blood
GBR60	La Mérica	2004	Tissue
GBR61	La Mérica	2004	Blood
GBR62	La Mérica	2004	Tissue
GBR63	La Mérica	2004	Tissue

* Founders (males and females) used for the breeding program.

Table S3. Log-normal priors (means/standard deviations, SD) and hyperpriors (means and variances for means and variances) used for MsVar analysis performed on *Gallotia bravoana* samples. N_O and N_1 are current and ancestral effective population sizes, respectively; Ө is the mutatio rate (μ) scaled in terms of N_O ($2N_0\mu$). Ta is the number of generations since expansion/decline.

Prior (mean/SD)	100 years	500 years	2500 years	10000 years
Log10 (N0)	3/3	3/3	3/3	3/3
Log10 (N1)	3/3	3/3	3/3	3/3
Log10 Ө	-3/1	-3/1	-3/1	-3/1
Log10 (Ta)	2/1	2.7/1	3.4/2	4/3

Hyperpriors (mean/SD/hiperpriors of mean/SD)				
Log10 (αN_0 σN_0 βN_0 τN_0)	6/2/0/0.5	6/2/0/0.5	6/2/0/0.5	6/2/0/0.5
Log10 (αN_1 σN_1 βN_1 τN_1)	6/2/0/0.5	6/2/0/0.5	6/2/0/0.5	6/2/0/0.5
Log10 (αμ σμ βμ τμ)	-3/0.25/0/0.5	-3/0.25/0/0.5	-3/0.25/0/0.5	-3/0.25/0/0.5
log10 (αTa σTa βTa τTa)	8/2/0/0.5	8/2/0/0.5	8/2/0/0.5	8/2/0/0.5
